# Comparative analysis of the *EGFR*, *HER2*, *c*-*MYC*, and *MET* variations in colorectal cancer determined by three different measures: gene copy number gain, amplification status and the 2013 ASCO/CAP guideline criterion for HER2 testing of breast cancer

**DOI:** 10.1186/s12967-017-1265-x

**Published:** 2017-08-01

**Authors:** Yoonjin Kwak, Sumi Yun, Soo Kyung Nam, An Na Seo, Kyu Sang Lee, Eun Shin, Heung-Kwon Oh, Duck Woo Kim, Sung Bum Kang, Woo Ho Kim, Hye Seung Lee

**Affiliations:** 1 Department of Pathology, Seoul National University Bundang Hospital, 173-82 Gumi-ro, Bundang-gu, Seongnam, Gyeonggi-do 463-707 Republic of Korea; 2 Department of Diagnostic Pathology, Samkwang Medical Laboratories, Seoul, Republic of Korea; 3 Department of Pathology, Kyungpook National University Hospital, Kyungpook National University School of Medicine, Daegu, Republic of Korea; 4 Department of Pathology, Veterans Health Service Medical Center, Seoul, Republic of Korea; 5 Department of Surgery, Seoul National University Bundang Hospital, Seongnam, Republic of Korea; 6 Department of Pathology, Seoul National University College of Medicine, Seoul, Republic of Korea

**Keywords:** Gene copy number variation, *EGFR*, *HER2*, *c*-*MYC*, *MET*, Colorectal cancer

## Abstract

**Background:**

The purpose of this study was to explore gene copy number (GCN) variation of *EGFR*, *HER2*, *c*-*MYC*, and *MET* in patients with primary colorectal cancer (CRC).

**Methods:**

Dual-colour silver-enhanced in situ hybridization was performed in tissue samples of 334 primary CRC patients. The amplification status (GCN ratio ≥2) and GCN gain (average GCN ≥4) data for the *EGFR*, *HER2*, *c*-*MYC* and *MET* genes were obtained. GCN variation was also assessed by the criterion of the 2013 ASCO/CAP guidelines for HER2 testing.

**Results:**

Amplification of *EGFR*, *HER2*, *c*-*MYC* and *MET* was detected in 8 (2.4%), 20 (6.0%), 29 (8.7%), and 14 (4.2%) patients, respectively. Of 66 patients with at least one amplified gene, five exhibited co-amplification of genes studied (*HER2*-*MET* co-amplification: two patients; *HER2*-*c*-*MYC* co-amplification: two patients; *EGFR*-*c*-*MYC* co-amplification: one patient). There were 109 patients with GCN gains of one or more genes (*EGFR*: 11/334, *HER2*: 29/334, *c*-*MYC*; 60/334, *MET*: 48/334) and 32.1% (35/109) had multiple GCN gains. When each GCN was assessed by the criterion of the ASCO/CAP 2013 guideline for HER2 testing, 116 people showed positive or equivocal results for one or more genes. The cumulative amplification status had no association with patients’ outcome. However, the cumulative results of the GCN gain and GCN status determined according to the ASCO/CAP guideline had a significant prognostic correlation in the univariate analysis (*P* values of 0.006 and 0.022, respectively). In the multivariate analysis, GCN gain and GCN status were independent prognostic factors (*P* values of 0.010 and 0.017, respectively).

**Conclusions:**

In this study, we evaluated GCN variation of four genes in a large sample of Korean CRC patients. The amplification status was not related to patient outcome. However, the GCN gain and GCN status according to the ASCO/CAP 2013 guideline were independent prognostic factors.

## Background

Gene copy number (GCN) variation, i.e., copy number difference in a genomic segment, occurs commonly and is one of the main mechanisms in shaping the human genome [1]. GCN variation has been found to play an important role in the stimulation of cell proliferation and decrease of apoptosis as well as in promoting development and progression of various cancers [1, 2]. Recent advancements in molecular genetics have made it possible to detect various GCN changes in human cancers [1, 2]. To date, many studies have reported the prognostic and predictive value of GCN variation in breast, lung, gastric and colorectal cancer (CRC) [3, 4]. Investigation of the molecular profiles of malignant cells may yield valuable prognostic information about each type of cancer. In addition, the sub-classification based on genetic alterations is also essential to select the patients who may benefit from drugs specifically targeted to a particular molecular alteration. Despite these clinical implications, the appropriate and standardized criterion to define positivity of each GCN variation remains uncertain, particularly in CRC. Therefore, further studies are required to develop the adequate criterion for the identification of genetic alterations and determination of reliable biomarkers.

CRC is known to be a heterogeneous disease with diverse molecular alterations including genetic changes in expression of *c*-*MYC* and *MET*, members of the human epidermal growth factor receptor (EGFR) family [5]. Human EGFR is a receptor kinase, which is a part of a complex signaling cascade that regulates cell proliferation and differentiation. EGFR is a representative member of the EGFR family. Dysregulation of EGFR expression is considered to be an important genetic alteration in the targeted treatment of advanced CRC. Several studies have assessed the prognostic value for overall survival of patients and predictive effect of the anti-EGFR treatment in CRC [6, 7]. However, the results of these studies remain unclear. This may be explained by the relatively small number of patients involved and discrepant cut-off values of GCN variation used in these studies.

The prognostic significance of changes in *HER2* (human epidermal growth receptor 2), *c*-*MYC* and *MET* is also uncertain. Previous studies of Kavanagh et al. [8] and Marx et al. [9] did not consider *HER2* gene amplification as a significant prognostic factor in CRC. In aforementioned studies, they used different cut-off value for determining the *HER2* genetic status; the *HER2* signal >4.0 [8] and the *HER2*/CEP17 ratio ≥2.0 [9]. However, there had been some controversy about the cut-off value and the prognostic significance of *HER2* gene alterations in CRC [10, 11]. In CRC, a number of studies have addressed the prognostic and predictive value of *c*-*MYC* and *MET* gene alterations applying different criteria, such as the target gene/corresponding CEP signal ratio of ≥2–3 for amplification and the target GCN gain of ≥4 copies [12–15]. Only few studies have been examined the GCN alteration of these genes with a criterion of the ASCO/CAP 2013 guideline for HER2 testing of breast cancer.

In this study, we analysed GCN variation of the *EGFR*, *HER2*, *c*-*MYC*, and *MET* genes in 334 colorectal cancer tissue samples using silver-enhanced in situ hybridization (SISH). In particular, we defined GCN variation according to several criteria and compared them with clinicopathological data and patient outcomes.

## Methods

### Patients and tissue samples

We examined 334 patients who underwent surgical resection of CRC tumours at the Seoul National University Bundang Hospital (Seongnam, South Korea) in the period between January 2005 and December 2006. The clinicopathological information and clinical follow-up data were obtained from the patients’ medical and pathological records. The patients who underwent preoperative chemotherapy or radiotherapy were excluded. The pathologic tumor-node-metastasis (pTNM) stage was defined according to the 7th edition of the American Joint Committee on Cancer (AJCC) staging system. The location of CRC was defined as follows: right colon (including caecum, ascending colon, hepatic flexure and transverse colon), left colon (including splenic flexure, descending colon and sigmoid colon) and rectum. Progression-free survival (PFS) and overall survival (OS) were defined as periods from the date of surgical treatment until the date of disease progression and the date of cancer-related death, respectively.

### Tissue microarray (TMA)

TMA was constructed using tissue samples with a 2-mm core diameter. The representative core areas of CRC specimens were obtained from the paraffin-embedded formalin-fixed tissue blocks and transferred into new TMA blocks, as previously described [16].

### Dual-colour silver-enhanced in situ hybridization

The genetic status of *EGFR*, *HER2*, *c*-*MYC*, and *MET* was evaluated by the dual-colour SISH technique. Briefly, consecutive unstained TMA slides were stained following the manufacturer’s protocol using the target gene DNA and corresponding CEP probes. The following probes were used: *EGFR* DNA and Chromosome 7 probes, *HER2* DNA and Chromosome 17 probes, *c*-*MYC* DNA and Chromosome 8 probes, and *MET* DNA and Chromosome 7 probes (Ventana Medical System, Tucson, AZ, USA). The target gene DNA and CEP probes were allowed to co-hybridize on the same slides and were visualized by the Ventana ultraView SISH detection kit on the Ventana BenchMark XT automated slide stainer. The target gene and corresponding CEP signals were detected as black and red signals, respectively.

### Evaluation of gene copy number variation

We interpreted the SISH signals in the hot spots of the target gene and corresponding CEP signals under 20× or 40× objectives. We counted the signals in each core on 60, 20, 50, and 100 overlapping tumour cell nuclei for the *EGFR*, *HER2 c*-*MYC* and *MET* genes, respectively. When there were clusters with many overlapping SISH signals, we counted the small clusters as six signals and large clusters—as twelve signals.

In the present study, the copy number status of the four genes was assessed by three different methods. Gene amplification was defined as the target gene per CEP signal ratio of ≥2.0 in counted tumour cell nuclei. To define gene copy number gain, we used a cut-off value of the average gene copy number being equal to or greater than 4. The gene copy number variation was also analysed according to the 2013 American Society of Clinical Oncology/College of American Pathologists (ASCO/CAP) HER2 testing criterion of breast cancer [17]. The result was considered to be positive if the target gene/CEP ratio was ≥2.0 or if the average gene copy number was ≥6.0 signals. The value of the target gene/CEP ratio <2.0 with an average gene copy number ≥4.0 and <6.0 signals was regarded as an equivocal result.

### Microsatellite instability (MSI) analysis

MSI analysis was carried out for each patient using a representative tumour region and corresponding normal regions. To evaluate the MSI status of CRC, a panel of five microsatellite markers including BAT-26, BAT-25, D5S346, D17S250, and S2S123 were analysed using autonomic sequencing according to the previously described protocol [12].

### Genomic data from the cancer genome atlas (TCGA)

We used the publicly available and downloaded genetic data set of aforementioned four genes from TCGA portal (http://cbioportal.org). The putative copy number alteration data was derived from Genome-Wide Human SNP Array 6.0 and analysed by the GISTIC 2.0 algorithm. The copy number alteration status was classified into five group; homozygous deletion, heterozygous deletion, neutral, gain, and high level amplification. Clinicopathologic parameters including MSI status were also obtained.

### Statistical analysis

Statistical analyses were performed using the SPSS software package, version 21.0 (IBM Corp., Armonk, NY, USA). The Chi square and Fisher’s exact tests were used to evaluate the association between the gene status and clinicopathological characteristics. The Kaplan–Meier analysis with the log-rank test was performed to determine the prognostic significance for overall survival, and multivariate Cox proportional hazard regression analysis was used to identify the independent prognostic factors. The results were considered to be statistically significant if *P* < 0.05.

## Results

### Patients and tumour characteristics

The patient group consisted of 185 men (55.4%) and 149 women (44.6%) with the mean age of 64.04 ± 11.57 years (range 20–95). CRC tumours located in the right colon, left colon, and rectum accounted for 93 (27.8%), 127 (38.0%) and 114 (34.1%) individual cases, respectively. Of these cases, 303 (90.7%) were low grade tumours and 31 (9.3%) cases were high grade tumours. The pTNM stage of resected specimens was classified according to the AJCC 7th edition. The distribution of pTNM stages was as follows: stage I—36 (10.8%); stage II—109 (32.6%); stage III—128 (38.3%); and stage IV—61 (18.3%). The results of the MSI analysis were available in 323 cases of which 296 (91.6%) cases were categorized as Microsatellite Stable/MSI-Low and 27 (8.4%) cases were categorized as MSI-High. Table 1 shows detailed clinicopathological characteristics of the CRC cases studied.

**Table 1:** Clinicopathologic features of 334 CRC patients

Characteristic	Examined no. (%)
Age (years)	
Mean ± SD	64.04 ± 11.57
Range	20–95
Tumor size (cm)	
Mean ± SD	5.32 ± 2.21
Range	1.0–13.0
Sex	
Male	185 (55.4)
Female	149 (44.6)
Location	
Right	93 (27.8)
Left	127 (38.0)
Rectum	114 (34.1)
Histologic grade	
Low grade	303 (90.7)
High grade	31 (9.3)
Tumor border	
Expanding	49 (14.7%)
Infiltrative	285 (85.3%)
Lymphatic invasion	
Absent	143 (42.8%)
Present	191 (57.2%)
Venous invasion	
Absent	272 (81.4%)
Present	62 (18.6%)
Neural invasion	
Absent	231 (69.2%)
Present	103 (30.8%)
pTNM stage	
I–III	273 (81.7)
IV	61 (18.3)
MSI status (n = 323)	
MSS/MSI-L	296 (91.6)
MSI-H	27 (8.4)

*SD* standard deviation, *MSI* microsatellite instability, *MSS* microsatellite instability stable, *MSI-L* microsatellite instability-low, *MSI-H* microsatellite instability-high

### Genetic status according to different criteria

**Fig. 1:**
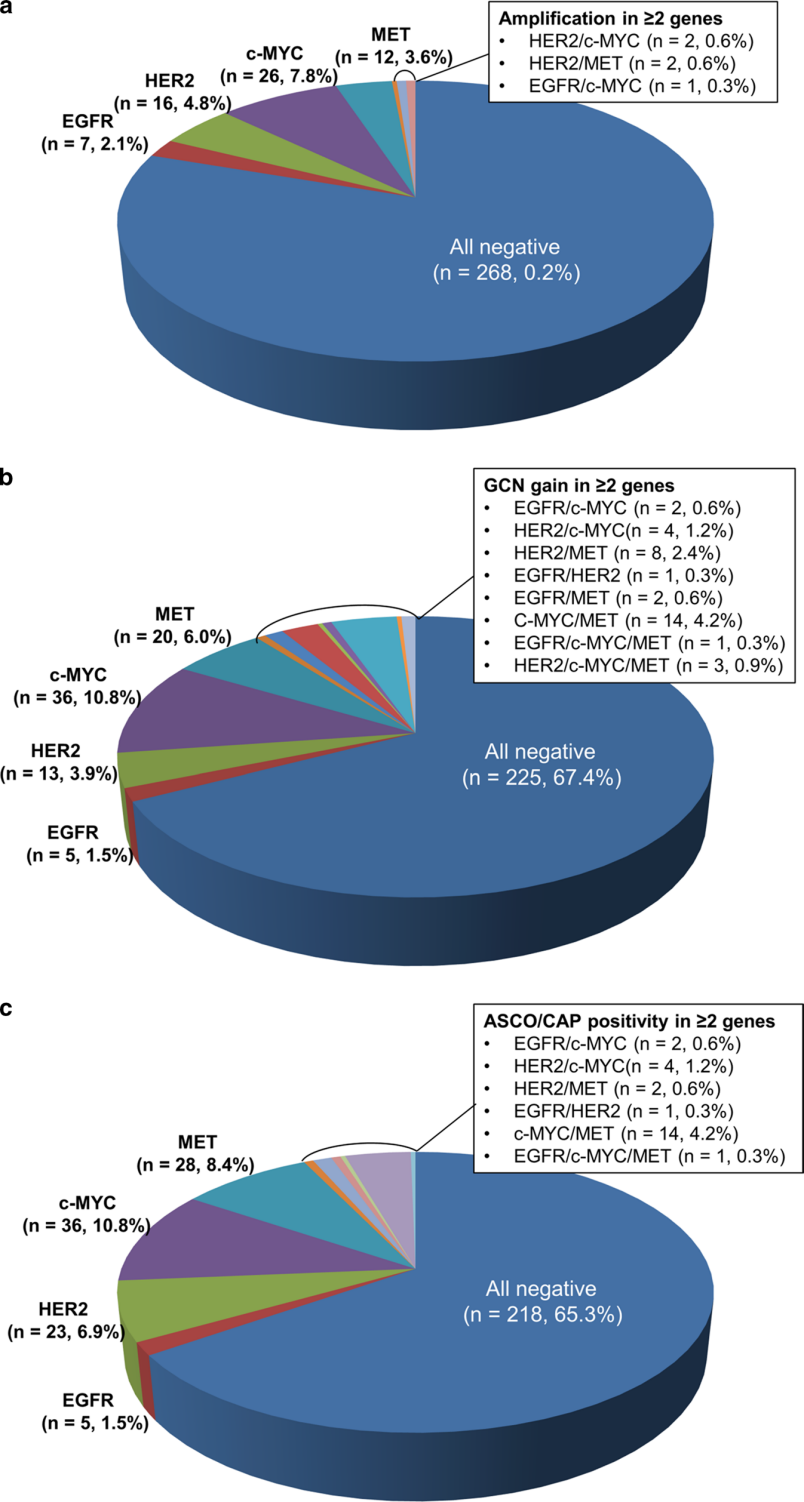
Distribution of *EGFR*, *HER2*, *c*-*MYC,* and *MET* genetic alterations according to (**a**) amplification data, (**b**) GCN gain results and (**c**) ASCO/CAP criterion

Among the 334 CRC cases, amplification of *EGFR*, *HER2*, *c*-*MYC,* and *MET* was observed in 8 (2.4%), 20 (6.0%), 29 (8.7%) and 14 (4.2%) cases, respectively. Amplification of these genes occurred in a mutually exclusive manner except for five cases, where co-amplification of two genes was observed. These five cases harboured *HER2/MET* co-amplification (two cases, 0.6%), *HER2/c*-*MYC* co-amplification (two cases, 0.6%), and *EGFR/c*-*MYC* co-amplification (one case, 0.3%). Application of a GCN gain cut-off revealed that 109 cases (32.6%) harboured GCN gain in ≥1 gene. Among these 109 cases, 32.1% (35/109) had multiple GCN gains in ≥2 genes. When the ASCO/CAP guideline was applied, 116 cases (34.7%) had positive or equivocal results. Furthermore, there were 35 cases (30.2%) of patients with equivocal or positive results in two and more genes. The detailed information on the genetic status of each of the four genes studied is described in Fig. 1.

### Association between the clinicopathological factors and genetic status

Next, we assessed the association between clinicopathological factors and genetic status of each of the four studied genes. *EGFR* amplification and GCN gain were not significantly associated with the tumour location, pathological stage, or MSI status (all *P* > 0.05). Using the ASCO/CAP criterion, *EGFR* positivity was significantly related to the pathological stage (*P* = 0.045). For the *HER2* gene, there was a trend towards association of *HER2* gene expression alterations with the tumour location in the rectum if any of the three criteria was used (*P* = 0.011, *P* = 0.073, and *P* = 0.010 for the amplification status, GCN gain and ASCO/CAP criterion, respectively). An association was also observed between *c*-*MYC* GCN gain and tumour location (*P* = 0.056, *P* = 0.042 and *P* = 0.083 for the amplification status, GCN gain and ASCO/CAP criterion, respectively). At the same time, *HER2* and *c*-*MYC* alterations were not related to the pathological stage or MSI status (all *P* > 0.05). In addition, *MET* GCN gain and positivity according to the ASCO/CAP criterion showed an association with the pathological stage (*P* = 0.003 and *P* = 0.020, respectively). The genetic alterations in *EGFR*, *HER2*, *c*-*MYC*, and *MET* assessed by three different criteria were almost mutually exclusive with the MSI-H status, as expected (Table 2) but the effects did not reach statistical significance (all *P* > 0.05).

**Table 2:** Correlation of genetic alteration according to the various criteria

Characteristics	Amplification			GCN gain			ASCO/CAP criteria			
	Negative	Positive	*P*	CN <4	4≤ CN	*P*	Negative	Equivocal	Positive	*P*
EGFR										
Location			1.000			0.803				0.419
Right colon	91 (97.8%)	2 (2.2%)		89 (95.7%)	4 (4.3%)		89 (95.7%)	2 (2.2%)	2 (2.2%)	
Left colon	124 (97.6%)	3 (2.4%)		123 (96.9%)	4 (3.1%)		123 (96.9%)	0 (0%)	4 (3.1%)	
Rectum	111 (97.4%)	3 (2.6%)		111 (97.4%)	3 (2.6%)		111 (97.4%)	0 (0%)	3 (2.6%)	
pTNM stage			1.000			0.429				0.045
I–III	266 (97.4%)	7 (2.6%)		265 (97.1%)	8 (2.9%)		265 (97.1%)	0 (0%)	8 (2.9%)	
IV	60 (98.4%)	1 (1.6%)		58 (95.1%)	3 (4.9%)		58 (95.1%)	2 (3.3%)	1 (1.6%)	
MSI status (n = 323)			1.000			0.609				1.000
MSS/MSI-L	288 (97.3%)	8 (2.7%)		285 (96.3%)	11 (3.7%)		285 (96.3%)	2 (0.7%)	9 (3.0%)	
MSI-H	27 (100%)	0 (0%)		27 (100%)	0 (0%)		27 (100%)	0 (0%)	0 (0%)	
HER2										
Location			0.011			0.073				0.015
Right colon	90 (96.8%)	3 (3.2%)		89 (95.7%)	4 (4.3%)		89 (95.7%)	1 (1.1%)	3 (3.2%)	
Left colon	123 (96.9%)	4 (3.1%)		117 (92.1%)	10 (7.9%)		116 (91.3%)	7 (5.5%)	4 (3.1%)	
Rectum	101 (88.6%)	13 (11.4%)		99 (86.8%)	15 (13.2%)		99 (86.8%)	2 (1.8%)	13 (11.4%)	
pTNM stage			1.000			0.248				0.422
I–III	256 (93.8%)	17 (6.2%)		247 (90.5%)	26 (9.5%)		246 (90.1%)	10 (3.7%)	17 (6.2%)	
IV	58 (95.1%)	3 (4.9%)		58 (95.1%)	3 (4.9%)		58 (95.1%)	0 (0%)	3 (4.9%)	
MSI status (n = 323)			1.000			0.491				1.000
MSS/MSI-L	278 (93.9%)	18 (6.1%)		269 (90.9%)	27 (9.1%)		268 (90.5%)	10 (3.4%)	18 (6.1%)	
MSI-H	26 (96.3%)	1 (3.7%)		26 (96.3%)	1 (3.7%)		26 (96.3%)	0 (0%)	1 (3.7%)	
c-MYC										
Location			0.056			0.042				0.083
Right colon	88 (94.6%)	5 (5.4%)		82 (88.2%)	11 (11.8%)		82 (88.2%)	6 (6.5%)	5 (5.4%)	
Left colon	110 (86.6%)	17 (13.4%)		96 (75.6%)	31 (24.4%)		96 (75.6%)	13 (10.2%)	18 (14.2%)	
Rectum	107 (93.9%)	7 (6.1%)		96 (84.2%)	18 (15.8%)		96 (84.2%)	11 (9.6%)	7 (6.1%)	
pTNM stage			0.723			0.136				0.199
I–III	250 (91.6%)	23 (8.4%)		228 (83.5%)	45 (16.5%)		228 (83.5%)	21 (7.7%)	24 (8.8%)	
IV	55 (90.2%)	6 (9.8%)		46 (75.4%)	15 (24.6%)		46 (75.4%)	9 (14.8%)	6 (9.8%)	
MSI status (n = 323)			0.491			0.061				0.155
MSS/MSI-L	269 (90.9%)	27 (9.1%)		240 (81.1%)	56 (18.9%)		240 (81.1%)	28 (9.5%)	28 (9.5%)	
MSI-H	26 (96.3%)	1 (3.7%)		26 (96.3%)	1 (3.7%)		26 (96.3%)	0 (0%)	1 (3.7%)	
MET										
Location			0.427			0.602				0.536
Right colon	87 (93.5%)	6 (6.5%)		77 (82.8%)	16 (17.2%)		75 (80.6%)	10 (10.8%)	8 (8.6%)	
Left colon	122 (96.1%)	5 (3.9%)		109 (85.8%)	18 (14.2%)		106 (83.5%)	15 (11.8%)	6 (4.7%)	
Rectum	111 (97.4%)	3 (2.6%)		100 (87.7%	14 (12.3%)		99 (86.8%)	11 (9.6%)	4 (3.5%)	
pTNM stage			1.000			0.003				0.020
I–III	261 (95.6%)	12 (4.4%)		241 (88.3%)	32 (11.7%)		235 (86.1%)	23 (8.4%)	15 (5.5%)	
IV	59 (96.7%)	2 (3.3%)		45 (73.8%)	16 (26.2%)		45 (73.8%)	13 (21.3%)	3 (4.9%)	
MSI status (n = 323)			0.609			0.147				0.298
MSS/MSI-L	284 (95.9%)	12 (4.1%)		252 (85.1%)	44 (14.9%)		247 (83.4%)	33 (11.1%)	16 (5.4%)	
MSI-H	27 (100%)	0 (0%)		26 (96.3%)	1 (3.7%)		26 (96.3%)	1 (3.7%)	0 (0%)	

*MSI* microsatellite instability, *MSS* microsatellite instability stable, *MSI-L* microsatellite instability-low, *MSI-H* microsatellite instability-high, *GCN* gene copy number, *CN* copy number, *ASCO/CAP* American Society of Clinical Oncology/College of American Pathologists

### Patient outcome according to the genetic status of *EGFR*, *HER2*, *c*-*MYC* and *MET*

**Fig. 2:**
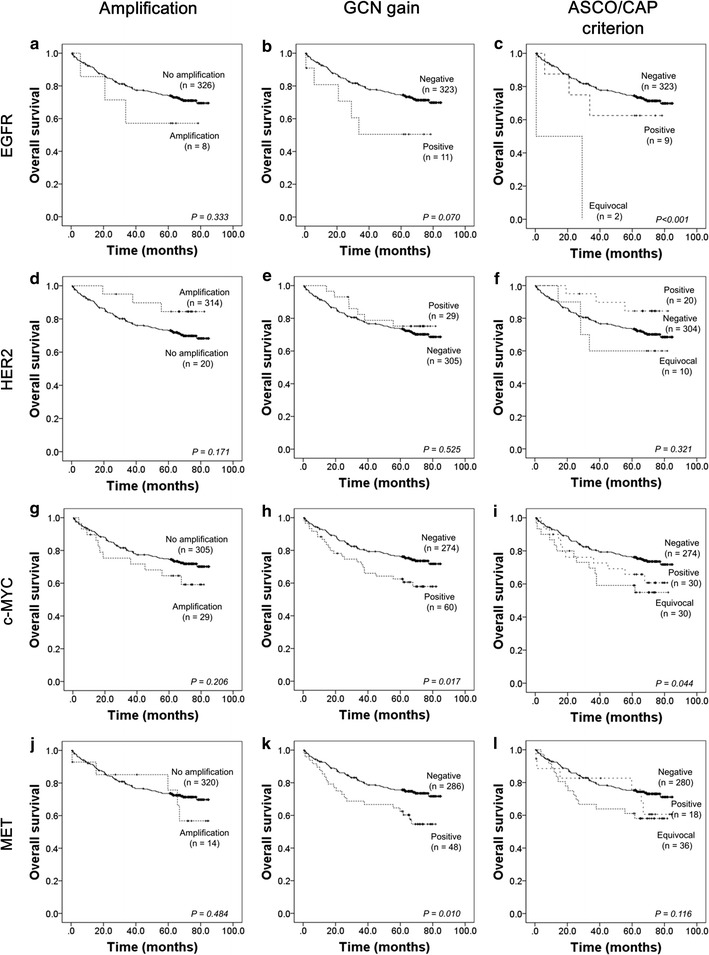
Kaplan–Meier survival analysis of overall survival of CRC patients with *EGFR*, *HER2*, *c*-*MYC,* and *MET* GCN changes, which were defined according to the following criteria: **a**, **d**, **g**, **j** amplification; **b**, **e**, **h**, **k** GCN gain and **c**, **f**, **i**, **l** ASCO/CAP criterion

To determine and compare the prognostic significance of *EGFR*, *HER2*, *c*-*MYC,* and *MET* alterations according to the three criteria defined above, survival analysis was performed using follow-up data collected for all patients. The median follow-up period comprised 66.1 months (range 0.6–85.1 months). As shown in Fig. 2, CRC patients with *c*-*MYC* and *MET* GCN gains, determined after the application of the GCN gain cut-off value as defined above, showed shorter OS compared to the patient group without these genetic changes (*P* = 0.017 and *P* = 0.010, respectively). The *EGFR* GCN gain tended to be associated with poor prognosis but the effect did not reach statistical significance (*P* = 0.070). When we applied the ASCO/CAP criterion, we observed statistically significant effects of the *EGFR* and *c*-*MYC* alterations on OS. The prognosis for the *EGFR* positive or equivocal CRC group was worse than for the patients from the *EGFR* negative CRC group (*P* < 0.001). Patients with a positive *c*-*MYC* result also had significantly shorter OS (*P* = 0.044). Interestingly, when the ASCO/CAP testing criterion was applied, patients with equivocal results for these genes tended to have the worst prognosis in our cohort (Fig. 2).

### Cumulative analysis of the effect of *EGFR*, *HER2*, *c*-*MYC* and *MET* alterations on overall survival

**Fig. 3:**
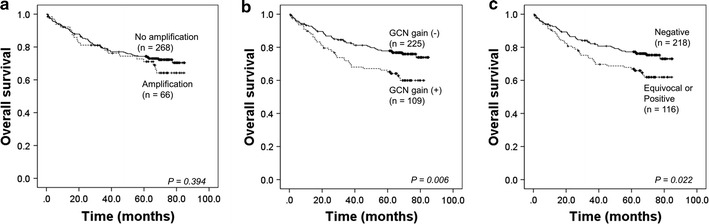
Cumulative survival analysis of overall survival of CRC patients with *EGFR*/*HER2*/*c*-*MYC*/*MET* GCN alteration, which were defined according to the following criteria: **a** amplification; **b** GCN gain; **c** ASCO/CAP criterion

We also performed the cumulative analysis of *EGFR, HER2, c*-*MYC* and *MET* status defined by these three criteria. CRC patients with amplification of any of these genes were found in 19.8% (66/334) of cases. GCN gain was observed in 32.6% (109/334). Equivocal or positive results according to the ASCO/CAP guideline were detected in 34.7% (116/334) cases (Table 3). In the Kaplan–Meier survival analysis, CRC cases with GCN gain was significantly associated with poor OS (*P* = 0.006). Also, the patients with equivocal or positive results defined by the ASCO/CAP criterion in any of the four genes were had worse outcome (*P* = 0.022). However, amplification of any of these genes was not associated with OS (*P* = 0.394) (Fig. 3). Multivariate analysis proved that genetic alterations of *EGFR, HER2, c*-*MYC,* or *MET* defined by the GCN gain or the ASCO/CAP criterion were significant unfavourable prognostic factors of OS (HR, 1.750 95% CI, 1.145–2.673; *P* = 0.010 and HR, 1.676 95% CI, 1.097–2.561; *P* = 0.017, respectively) (Table 4).

**Table 3:** Combined clinicopathologic analysis according to the various criteria

Characteristics	Amplification			GCN gain			2013 ASCO/CAP guideline		
	Negative	Positive	*P*	CN< 4	4 ≤CN	*P*	Negative	Equivocal or positive	*P*
Location			0.403			0.241			0.213
Right colon	79 (84.9%)	14 (15.1%)		69 (74.2%)	24 (25.8%)		67 (72.0%)	26 (28.0%)	
Left colon	100 (78.7%)	27 (21.3%)		81 (63.8%)	46 (36.2%)		77 (60.6%)	50 (39.4%)	
Rectum	89 (78.1%)	25 (21.9%)		75 (65.8%)	39 (34.2%)		74 (64.9%)	40 (35.1%)	
pTNM stage			0.708			0.124			0.257
I–III	218 (79.9%)	55 (20.1%)		189 (69.2%)	84 (30.8%)		182 (66.7%)	91 (33.3%)	
IV	50 (82.0%)	11 (18.0%)		36 (59.0%)	25 (41.0%)		36 (59.0%)	25 (41.0%)	
MSI status (n = 323)			0.097			0.004			0.002
MSS/MSI-L	235 (79.4%)	61 (20.6%)		194 (65.5%)	102 (34.5%)		188 (63.5%)	108 (36.5%)	
MSI-H	25 (92.6%)	2 (7.4%)		25 (92.6%)	2 (7.4%)		25 (92.6%)	2 (7.4%)	

*MSI* microsatellite instability, *MSS* microsatellite instability stable, *MSI-L* microsatellite instability-low, *MSI-H* microsatellite instability-high, *GCN* gene copy number, *CN* copy number, *ASCO/CAP* American Society of Clinical Oncology/College of American Pathologists

**Table 4:** Univariate and multivariate analysis according to the various criteria

Factors	Univariate analysis	*P*
Age	1.022 (1.033–1.042)	0.021
Sex	0.823 (0.545–1.245)	0.357
Tumor size	1.150 (1.058–1.251)	0.001
Histologic grade (high vs. low)	3.582 (2.136–6.007)	<0.001
Tumor border (infiltrative vs. expanding)	4.173 (1.532–11.365)	0.005
Lymphatic invasion (present vs. absent)	3.345 (2.039–5.488)	<0.001
Venous invasion (present vs. absent)	3.777 (2.484–5.741)	<0.001
Neural invasion (present vs. absent)	3.601 (2.389–5.427)	<0.001
pTNM stage (IV vs. I–III)	7.727 (5.109–11.687)	<0.001
Amplification in any gene (≥2 vs. <2)	1.235 (0.759–2.008)	0.395
GCN gain in any gene (≥4 vs. <4)	1.770 (1.175–2.666)	0.006
ASCO/CAP criteria in any gene (positive/equivocal vs. negative)	1.608 (1.067–2.421)	0.023

Factors	Multivariate analysis	*P*
GCN gain		
Age	1.038 (1.015–1.061)	0.001
Sex	0.643 (0.414–0.999)	0.049
Tumor size	1.115 (1.001–1.243)	0.048
Histologic grade (high vs. low)	2.843 (1.571–5.143)	0.001
Tumor border (infiltrative vs. expanding)	2.550 (0.899–7.231)	0.078
Lymphatic invasion (present vs. absent)	1.296 (0.737–2.276)	0.368
Venous invasion (present vs. absent)	1.197 (0.720–1.989)	0.489
Neural invasion (present vs. absent)	2.400 (1.506–3.827)	<0.001
pTNM stage (IV vs. I–III)	4.791 (2.891–7.940)	<0.001
GCN gain in any gene (≥4 vs. <4)	1.750 (1.145–2.673)	0.010
ASCO/CAP criteria		
Age	1.038 (1.016–1.061)	0.001
Sex	0.645 (0.415–1.003)	0.052
Tumor size	1.114 (0.999–1.241)	0.052
Histologic grade (high vs. low)	2.857 (1.578–5.174)	0.001
Tumor border (infiltrative vs. expanding)	2.525 (0.891–7.155)	0.081
Lymphatic invasion (present vs. absent)	1.289 (0.734–2.265)	0.377
Venous invasion (present vs. absent)	1.197 (0.721–1.987)	0.488
Neural invasion (present vs. absent)	2.414 (1.514–3.850)	<0.001
pTNM stage (IV vs. I–III)	4.868 (2.941–8.057)	<0.001
ASCO/CAP criteria in any gene (positive/equivocal vs. negative)	1.676 (1.097–2.561)	0.017

*MSI* microsatellite instability, *GCN* gene copy number, *ASCO/CAP* American Society of Clinical Oncology/College of American Pathologists

### Analysis of TCGA data

**Fig. 4:**
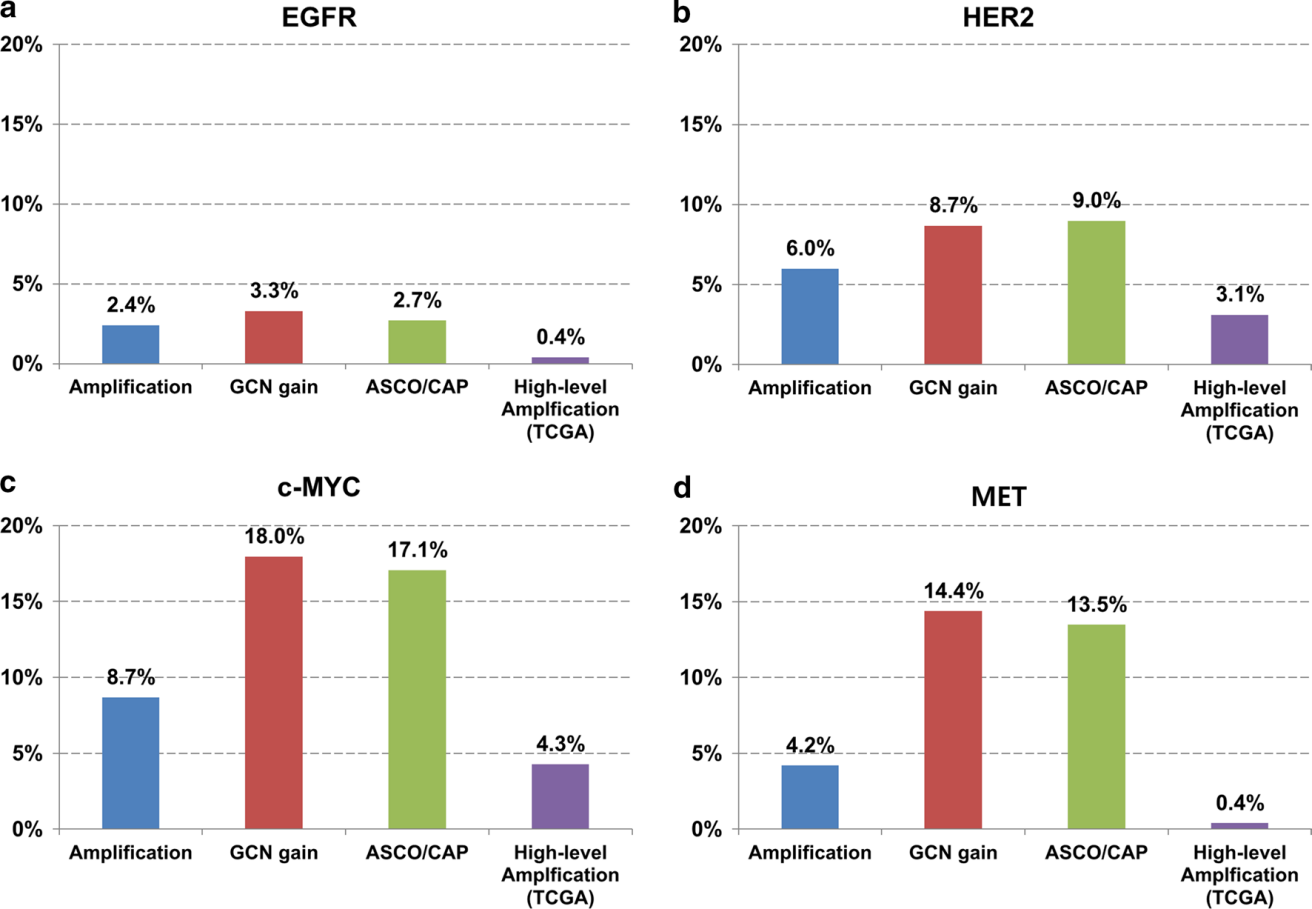
Comparison of the rate of GCN alteration obtained by in situ hybridization methods and array-based platform: **a** EGFR, **b** HER2, **c** c-MYC, and **d** MET

A total of 257 cases of CRC were used for comparative analysis. High-level amplification of *EGFR*, *HER2*, *c*-*MYC,* and *MET* was observed in 1 (0.4%), 8 (3.1%), 11 (4.3%) and 1 (0.4%) cases, respectively (Fig. 4). There was no case had co-amplification in TCGA cohort. The amplification rate of each gene was slightly lower than the rate showed in 334 Korean CRC cohort. Copy number gain of *EGFR*, *HER2*, *c*-*MYC,* and *MET* was observed in 114 (44.4%), 40 (15.6%), 119 (46.3%) and 103 (40.1%) cases, respectively. Of 257 cases, 128 (49.8%) harboured GCN gain in ≥1 gene.

We analyzed the association between clinicopathological factors and genetic status of the four genes using TCGA data in like manner used for Korean CRC cohort. Similar to the results of Korean CRC cohort, there was no statistical significance between the status of high-level amplification in ≥1 gene and MSI status (P = 0.326). The status of GCN gain in ≥1 gene was significantly correlated with MSI-H status (P < 0.001). Other clinicopathological parameters including pathologic stage had no significant correlation with gene copy number status of four genes (data not shown).

None of the copy number alteration status of each four genes had association with OS. In cumulative analysis, there was no significant difference of OS between the patients with high-level amplification of any of four genes or patients with no amplification (P = 0.704). The status of GCN gain in ≥1 gene also had no prognostic correlation (P = 0.691).

## Discussion

Much effort has been focused on improving CRC treatment strategies, and a number of genetic alterations associated with CRC development have been evaluated. Aberrant activation of *EGFR, HER2, c*-*MYC*, or *MET* is considered to be a promising target for specific molecular treatments in various cancers, including CRC [7, 12, 15, 18]. Although many studies suggested a prognostic and predictive impact of such genetic alterations, a standardized criterion for them in CRC has not been established. In this study, we evaluated the genetic status of *EGFR, HER2, c*-*MYC* and *MET* in 334 CRC samples and its association with patients’ prognosis in 334 CRC samples using dual-colour SISH analysis. We evaluated the applicability of the ASCO/CAP HER2 guideline criterion for breast cancer to assess the *EGFR, HER2, c*-*MYC,* and *MET* gene status. We also assessed GCN gain and amplification of these genes in the CRC samples. We found that comparative analysis of the genetic status by using several criteria supported the conclusion that GCN gain and GCN result according to the ASCO/CAP criteria were independent prognostic factors in consecutive CRC patients.

Genetic alterations of *HER2* have been studied particularly actively in breast and gastric cancers [17, 19]. Currently, evaluation of the *HER2* status in breast cancer is included as part of a routine test according to the ASCO/CAP criteria. However, because many previous studies evaluated the genomic status of these genes in CRC by disparate criteria, the prognostic and predictive impact of the *HER2* status has been controversial. Our study is one of the few studies, in which genetic changes in CRC were assessed according to the 2013 ASCO/CAP criterion. When we applied the ASCO/CAP HER2 test criterion to CRC cases, *EGFR* and *c*-*MYC* positivity showed significant association with poor prognosis. In the cumulative analysis of *EGFR, HER2, c*-*MYC,* and *MET*, CRC cases with positive results for any of these genes was a significant predictor of poor overall survival. Although further studies that would evaluate this concordance with fluorescence in situ hybridization and immunohistochemistry methods are needed, our findings suggest that the 2013 ASCO/CAP HER2 testing criterion for breast cancer is applicable for determination of genetic changes in CRC.

We defined GCN gain as the signal equal or higher than the cut-off value of 4.0 for each gene signal per cell. In our results, GCN gain in the *EGFR*, *c*-*MYC*, and *MET* genes, determined according to this criterion, was significantly associated with patient survival. In the multivariate analysis, application of this cut-off value revealed that GCN gain in any of the four genes analysed was a significant poor prognosis factor in CRC, in line with the results obtained using the ASCO/CAP criterion. In addition, we defined the equivocal result according to the ASCO/CAP guideline, namely when 6> gene copy number ≥4.0 and found that for such CRC cases, the prognosis was worse than for those with negative or positive results. Thus, we demonstrated in our study that the cut-off value of ≥4.0 as well as the ASCO/CAP criterion could be applied as a reliable cut-off point to determine GCN gain as a molecular prognostic marker in CRC.

As has been published previously [6, 13, 14], we observed that the rates of *c*-*MYC* and *MET* amplification were relatively low. Interestingly, approximately 20–30% of genetic alterations in *c*-*MYC* and *MET* were detected using GCN gain and the ASCO/CAP criteria. The discrepancy was also found in the rates of CRC cases with multiple genetic changes. There were only 5 cases of CRC with amplifications of multiple genes. However, there were 35 cases of CRC accompanied by GCN gains and ASCO/CAP positivity in multiple genes. These genetic changes were considered to have clinical significance because they were associated with poor prognosis. Specific drugs targeting EGFR represent one of the main treatment strategies in advanced CRC patients [20, 21], and some studies reported the association between the *EGFR* GCN and resistance to anti-EGFR treatment [7, 22, 23]. Currently, the association between other genetic changes and the resistance to EGFR inhibitors is a subject of intense scientific scrutiny. Furthermore, attempts to exploit these genetic changes for targeted therapy are currently underway in several preclinical and clinical trials [18, 24, 25]. Based on this knowledge, the ability to detect the presence of these genetic changes in CRC has a great clinical significance. Our findings, therefore, suggest that GCN gain and the ASCO/CAP criterion are more useful to detect genetic alterations accurately.

In present study, we analysed somatic gene copy alteration of TCGA dataset and compare the results from that of Korean CRC cohort. The rate of high-level amplification of each gene using array-based method was lower than amplification rate obtained by SISH method. The proportion of MET gene amplification was highest and that of EGFR gene amplification was lowest among four genes in both cohorts. Correlation between copy number gain and MSI-H status was also observed in both cohorts. However the direct comparison of these results derived by different detection methods would be unsuitable.

Several platforms for detection of GCN alteration are currently available, but it is still unclear whether array-based platform is an ideal detection method. A lack of reproducibility and concordance for array-based method has been shown in previously published study by Pinto et al. [26]. Normal tissue contamination or unknown ploidies of tumor cells is another challenges for array-based platform. In HER2 testing for breast or gastric cancer, in situ hybridization method is the gold standard to assess patient’s further treatment [27]. In this study, the prognostic significance of GCN gain was only confirmed in Korean CRC when the genetic status analysed by GCN gain and ASCO/CAP guideline criterion. From the clinical viewpoint, detection of GCN alteration by classic in situ hybridization method could be more useful.

Despite several genes being proposed as prognostic markers based on the GCN gain or the ASCO/CAP criterion data, our results have some limitations. Some genetic alterations, including changes in *EGFR* and *MET*, were observed at relatively low frequencies, and these alterations were detected in less than 5% of cancers analysed in our study. Based on our results, evaluation of these genetic changes in large cohorts of samples using GCN gain or the ASCO/CAP guideline criterion is needed to determine their prognostic impact. Additionally, *EGFR, HER2, c*-*MYC,* and *MET* have been identified to be potential biomarkers that predict the efficacy of the pharmacological treatments targeted against protein products of these genes in various cancers. The determination and exact evaluation of cases with positive copy number changes in these genes may help in developing more effective treatment strategies by selecting patients who may benefit from the targeted treatments directed against the corresponding genetic changes. Further studies are necessary to explore if the GCN gain defined by the cut-off point of 4.0 and by the ASCO/CAP criterion has a predictive impact on the efficacy of treatments targeted to specific genetic changes in CRC. We analysed the genetic status using TMA, which may not be representative of the whole CRC tumour due to the use of only small cores taken from each specimen. Some previous studies also reported that cancers often had genomic heterogeneity [7, 9], so a small tissue core of TMA may not fully represent each cancer at the genetic level. Thus, we need efficient validation of the heterogeneity of these genetic alterations in CRC.

## Conclusions

We evaluated *EGFR, HER2, c*-*MYC*, and *MET* gene status by determining the degree of amplification, GCN gain and the 2013 ASCO/CAP HER2 testing guideline criterion for breast cancer. In the cumulative analysis of these genes, we revealed that genetic variation in any of four genes according to the GCN gain defined by the cut-off point of 4.0 or to the ASCO/CAP criterion was an independent prognostic factor in CRC.

## Abbreviations

GCN: gene copy number
CRC: colorectal cancer
*EGFR*: human epidermal growth factor receptor
*HER2*: human epidermal growth receptor 2
SISH: silver in situ hybridization
PFS: progression-free survival
OS: overall survival
TMA: tissue microarray
ASCO/CAP: American Society of Clinical Oncology/College of American Pathologists
MSI: microsatellite instability
